# Preparation of Magnetic Hemicellulosic Composite Microspheres and Adsorption of Copper Ions

**DOI:** 10.3390/polym16243460

**Published:** 2024-12-11

**Authors:** Muhammad Sheraz, Xiao-Feng Sun, Yongke Wang, Adeena Siddiqui, Jiayi Chen, Le Sun

**Affiliations:** 1 Research Centre of Advanced Chemical Engineering, School of Chemistry and Chemical Engineering, Northwestern Polytechnical University, Xi’an 710129, China; sherazmuhammad@mail.nwpu.edu.cn (M.S.);; 2 Shenzhen Research Institute, Northwestern Polytechnical University, Shenzhen 518063, China; 3 Faculty of Biosciences, Shaheed Zulfikar Ali Bhutto Institute of Science and Technology University, Karachi 75600, Pakistan

**Keywords:** hemicellulose, magnetic microspheres, preparation, adsorption property, Fe_3_O_4_ nanoparticles

## Abstract

In this study, the fabrication of magnetic hemicellulosic composite microspheres and the adsorption of copper ions are explored. The microspheres were prepared by the micro-emulsion technique, using Fe_3_O_4_ nanoparticles and hemicellulose extracted from wheat straw with the ionic liquid B[mim]Cl as a solvent. Fe_3_O_4_ nanoparticles, synthesized through coprecipitation, were evenly encapsulated within the hemicellulosic microspheres. The Fe_3_O_4_ nanoparticles measured 10–15 nm in size, while the microspheres had an average diameter of about 20 μm and displayed a saturation magnetization of 35.95 emu/g. The optimal conditions for copper adsorption by the microspheres were found to be a pH of 5.0, a temperature of 323 K, and an initial copper ion concentration of 80 mg/L, resulting in an adsorption capacity of 85.65 mg/g after 24 h. The adsorption kinetics followed a pseudo-second-order model, and the Langmuir isotherm suggested a monomolecular layer adsorption mechanism, with a theoretical maximum capacity of 149.25 mg/g. In summary, the magnetic hemicellulosic microspheres exhibited considerable adsorption potential and favorable recycling capabilities for copper ions.

## 1. Introduction

**Figure 1:**
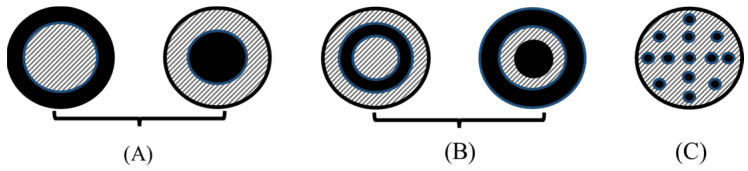
Common structure diagrams of magnetic polymer microspheres: (**A**) core-shell; (**B**) sandwich; and (**C**) hybrid.

In recent decades, magnetic polymer microspheres have attracted considerable interest and made significant progress. These microspheres are spherical composite materials, varying in size from nano- to microscale, and consist of polymer materials combined with magnetic particles. As early as the 1980s, researchers had successfully created polystyrene microspheres with excellent monodispersity, laying the groundwork for the development of polymer microsphere materials [1]. Magnetic polymer microspheres generally feature the following three primary structures: (A) core-shell, (B) sandwich, and (C) hybrid, as shown in Figure 1.

At present, a variety of types and structures of magnetic polymer microspheres have been developed, building on the basic core-shell design (with magnetic particles forming the core and polymer serving as the shell). These include multilayer, porous, flower-like, and Janus structures [2]. The notable characteristics of polymer microspheres include the following: (a) small size and volume; (b) large specific surface area; (c) diffusion capability; (d) stable dispersion; (e) uniform size, surface chemistry, and morphology; and (f) variability in size, surface chemistry, and morphology [3].

Monomer polymerization is the most extensively studied and commonly used method for fabricating magnetic polymer microspheres with a core-shell structure. This approach can be categorized into the three specific processes of suspension polymerization [4], dispersion polymerization [5], and emulsion polymerization [6]. The suspension polymerization system consists of monomers, initiator, stabilizer, and an aqueous phase. The intense mechanical shear breaks the monomer phase into small droplets, where free radical polymerization occurs. Suspension polymerization is favored in the production of microspheres of a larger size [7]. One of the relatively easy methods of preparation of microspheres is the dispersion polymerization technique developed in the 1970s. The monomer, initiator, stabilizer, and solvent are mixed in a homogeneous solution. The stabilizer usually can supply either steric or electrostatic stability and enables the fabrication of microspheres with uniform particle sizes [8]. In emulsion polymerization, water is used as the continuous phase. Using an emulsifier or surfactant, and under the appropriate agitation conditions, the distribution of the monomer within the aqueous phase is via micelles, in which it becomes solubilized and dissolved. The initiator, at a specified temperature, generates free radicals which initiate polymerization or co-polymerization inside the micelle. The resulting polymers are fine particles, dispersed in water solutions, and stabilized with soaps and, in certain cases, protective colloids. Emulsion polymerization possesses several characteristic features—a high rate of polymerization, high molecular weight of the product, stability of the dispersed system, and ease of control—rendering the process suitable for continuous operation [9,10]. In addition, some other established approaches for the preparation of magnetic polymer microspheres include surface imprinting [11] and solvent evaporation methods [12].

Magnetic particles used in these polymer microspheres are Fe_3_O_4_, γ-Fe_2_;O_3_, Fe, Co, Ni, and their respective compounds. Of these, Fe_3_O_4_ nanoparticles are mainly preferred because they show superior performances and the techniques for their preparation are well established. The Fe^2+^ and Fe^3+^ ions inside a unit cell are arranged without regularity, and electrons can migrate quickly; hence, they offer intermediate electrical conductivity [13]. Additionally, Fe_3_O_4_ has a very high saturated magnetization of 92 emu/g at room temperature [14]. Several common synthesis techniques of Fe_3_O_4_ nanoparticles included the coprecipitation method [15], hydrothermal method [16], microemulsion method [17], microwave-assisted synthesis [18], and ultrasonic methods [19]. Because of the above-mentioned properties and the relatively well-developed preparation methods, the Fe_3_O_4_ nanoparticles have been widely applied in the fields of biological engineering [20], medical engineering [21], and environmental science [22].

Currently, the natural polymer materials that involve the preparation of magnetic composite microspheres mainly include chitosan [23], cellulose [24], and modified cellulose [25]. However, the use of hemicellulose as a raw material for creating magnetic composite microspheres is less commonly reported in the literature. Hemicellulose is a widely available polymer found in nature, making up about 30–35% of the total lignocellulosic biomass, which makes it the second most abundant polysaccharide after cellulose. It has many advantages, including availability, cost-effectiveness, environmental friendliness, renewability, biodegradability, and non-toxicity, all of which contribute to its important role in various applications [26]. Hemicellulose can be extracted through various methods, such as hydrothermal processes [27,28], ionic liquid method [29], ultrasonic-assisted extraction [30], and biological refining method [31]. Presently, hemicellulose finds applications in numerous fields, including biological engineering [32], chemical engineering [33], biomedical applications [34], and environmental applications [33,35]. In the food industry, it plays several roles, functioning as an adhesive, thickener, stabilizer, hydrogel, dietary fiber, film-forming agent, and emulsifier [36].

Magnetic polymer microspheres, due to their outstanding physical and chemical properties, are ideal for a variety of applications. These applications encompass cell separation in biological engineering [37], the separation and purification of nucleic acids [38,39], protein delivery [40], and enzyme immobilization [41]. They are also employed in medical engineering [42], environmental science [43,44], and food analysis [45]. Microspheres offer major advantages in heavy metal adsorption because of the uniformly sized beads, a very high surface area-to-volume ratio, and the mesoporous structure that enables efficient binding and rapid adsorption kinetics. The spherical shape guarantees uniform interaction with adsorbates that promotes access to active sites and enhances the final adsorption performance [46]. Moreover, microspheres help to enhance dispersion stability in solutions as they can avoid aggregation and maintain stable performance during the adsorption process. They can be also modified to carry different functional groups or coatings, concentrating on specific ions such as copper, which improves the specificity and selectivity of the adsorption process [47]. For example, studies have shown that functionalized magnetic microspheres provide excellent adsorption capabilities, combining high efficiency and ease of magnetically induced separation. Therefore, this approach simplifies recovery and reuse, rendering microspheres highly favorable for applications in environmental remediation and water treatment [48].

In this study, the creation of hemicellulose/Fe_3_O_4_ magnetic composite microspheres for the adsorption of copper ions is motivated by their efficiency, ease of separation, environmental benefits, versatility, cost-effectiveness, and potential. These factors collectively make magnetic hemicellulosic microspheres a promising solution for addressing the challenges associated with heavy metal contamination in various industries and ecosystems. Fe_3_O_4_ nanoparticles were produced through the coprecipitation method, and the magnetic hemicellulosic microspheres were effectively prepared using the micro-emulsion technique. The characterization methods used in the present analysis included FT-IR, SEM, TEM, magnetic measurement, and particle size analysis. This study examined the optimum conditions of copper ions adsorption, adsorption kinetics and thermodynamics to determine the adsorption capacity and reusability of the hemicellulose/Fe_3_O_4_ magnetic microspheres.

## 2. Materials and Methods

### 2.1. Reagents

The reagents used in this study included ferric chloride hexahydrate (FeCl_3_·6H_2_;O), iron(II) chloride tetrahydrate (FeCl_2_;·4H_2_;O), N-methylimidazole, 1-chlorobutane, anhydrous ethanol, copper sulfate (CuSO_4_), sodium hydroxide (NaOH), sodium diethyldithiocarbamate, hydrochloric acid (HCl), ethylenediaminetetraacetic acid (EDTA), hydrogen peroxide (H_2_;O_2_;, 30%), ammonium hydroxide (NH_3_·H_2_;O), acetone, vacuum oil, and Tween 80, which were provided by Tianjin Jinbei Fine Chemical Co., Ltd. (Tianjin, China). Hemicellulose is extracted from wheat straw, which is comprised of 85.3% xylose and 9.8% arabinose determined by gas chromatography.

### 2.2. Preparation of Hemicellulose/Fe_3_O_4_ Magnetic Composite Microspheres

#### 2.2.1. Synthesis of Fe_3_O_4_ Nanoparticles

In this work, magnetic Fe_3_O_4_ nanoparticles were prepared by the co-precipitation method [49]. The mixed solution of 50 mL (0.2 mol/L) FeCl_3_·6H_2_O and 50 mL (0.1 mol/L) FeCl_2_·4H_2_O in a 200 mL three-necked flask was stirred mechanically (600 r/min) for 0.5 h at 80 °C under nitrogen atmosphere. NH_3_·H_2_O (1.5 mol/L) was dropped into the solution slowly until the pH reached a level of 8 and was stirred for 2 h. Following this, 5 mL of oleic acid was introduced to the solution, and the reaction was allowed to proceed for 3 h. The resulting black Fe_3_O_4_ particles were separated using a magnet, washed several times with ethanol and distilled water, and then dried in a vacuum oven at 60 °C for 24 h. Finally, the magnetic Fe_3_O_4_ nanoparticles were obtained after grinding.

#### 2.2.2. Synthesis of Ionic Liquid 1-Butyl-3-Methylimidazolium Chloride (B[mim]Cl)

**Figure 2:**
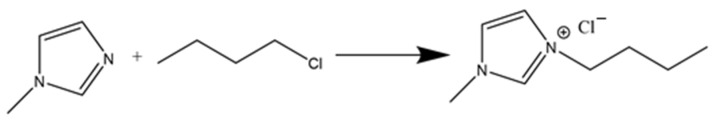
Synthesizing reaction of ionic liquid B[mim]Cl.

Butyl-3-methylimidazolium chloride (B[mim]Cl) was synthesized via a direct synthesis method (Figure 2). Under nitrogen protection, 1 mol of N-methylimidazole and 1 mol of 1-chlorobutane were dissolved in 200 mL of acetonitrile in a 500 mL three-necked flask and refluxed for 48 h at 80 °C. After cooling to room temperature, the residual substances were removed by distillation under reduced pressure. The remaining material was washed repeatedly with a mixture of acetonitrile and ethyl acetate (1:5), yielding a pale-yellow viscous liquid, which was the target compound.

#### 2.2.3. Preparation of Composite Microspheres

The microspheres were prepared by the micro-emulsion technique, using Fe_3_O_4_ nanoparticles and hemicellulose extracted from wheat straw with the ionic liquid B[mim]Cl as a solvent [50]. Firstly, hemicellulose (6 wt.%) was dissolved in ionic liquid under heating and stirring at 100 °C for 1 h to obtain a pale-yellow colloidal solution. Under vigorous stirring for 30 min, Fe_3_O_4_ nanoparticles were dispersed into the solution at a mass of one-third of hemicellulose. The mixture was emulsified in a system comprising vacuum pump oil and Span 80 under high-speed stirring at 1000 rpm for 3 h. Then, 25 mL of absolute ethanol was slowly added, and the suspension was cooled to room temperature. The microspheres were separated magnetically, washed thrice with ethanol and distilled water, and finally freeze-dried.

### 2.3. Characterization Methods

For Fourier transform infrared (FT-IR) analysis, the magnetic hemicellulosic microspheres were ground, and a certain amount of the sample was mixed with dried potassium bromide (KBr) which was operating as a transparent medium, and KBr allowed IR light through the mixture for clear spectral analysis [51]. The mixture was pressed into a pellet in mold and analyzed with an FTIR spectrometer (TENSOR 27, Bruker Corporation, Karlsruhe, Germany) in the scanning range of 400–4000 cm^−1^. For surface morphology analysis, the microspheres were sputtered with a thin layer of gold, and these were observed using scanning electron microscope (SEM) (Verios G4, FEI Company, Hillsboro, OR, USA) at an accelerating voltage of 20 kV. Internal structure evaluation was performed using transmission electron microscopy (TEM) (Themis Z, FEI Company, USA) by dispersing the powder sample in acetone, subjecting it to ultrasonic treatment at 40 W for 30 min, and depositing the suspension onto a copper grid for observation at an operating voltage of 75 kV. The magnetic properties were studied using a vibrating sample magnetometer (Model 7410; Lake Shore Cryotronics Inc., Westerville, OH, USA). The microsphere size distribution was determined using a laser particle size analyzer (PSA 1190; Anton Paar, Graz, Austria) after the sample was ultrasonically dispersed in an ultrasonic bath (Shenzhen Yunjiao Technology Co., Ltd., Shenzhen, China) at 40 W in deionized water. Unless otherwise stated, the experiments were conducted at room temperature.

### 2.4. Adsorption Experiments

The standard analysis curve of copper ion concentration was established using a UV–visible spectrophotometer (Jingke 752N; Shanghai, China). The absorbance was recorded as *A*, and *C* was the concentration of copper ion. The adsorption property of the magnetic hemicellulosic microspheres for copper ions was studied, and the effects of pH, adsorption temperature, and the initial concentration of Cu^2+^ (C_0_) on the adsorption performance were also investigated. Finally, the adsorption isotherm, thermodynamics, and kinetics of copper ions on the magnetic hemicellulosic microspheres were also studied, as well as the recyclability of the microspheres was also tested.

#### 2.4.1. Determination of Adsorption Capacity of the Microspheres for Copper Ions

To test the adsorption capacity of the microspheres, 20 mg of magnetic hemicellulosic microspheres were added to a 100 mL stopper Erlenmeyer flask that contained a copper ion solution with a known concentration and pH. The flask was placed on a temperature-controlled shaker at the specified temperature and oscillation frequency for 24 h. After reaching adsorption equilibrium, a magnet was used to separate the magnetic hemicellulosic microspheres from the solution. The absorbance of the supernatant was measured using a UV spectrophotometer, and this enabled the calculation of the concentration of copper ions in the sample based on the previously established standard curve. The adsorption amount (q) of copper ions by the magnetic hemicellulosic microspheres was calculated by Equation (1):

(1) $$q=\frac{(C_{0}-C_{t})\times V_{t}}{M}$$

In this context, *q* (mg/g) refers to the adsorption amount of the magnetic hemicellulosic microspheres for copper ions. The parameters are defined as follows: *C*_0_ (mg/mL) represents the initial concentration of Cu^2+^ in the solution, while *C*_*t*_ (μg/mL) indicates the concentration of Cu^2+^ in the selected sample solution at the adsorption equilibrium *t*. Additionally, *V*_*t*_ (mL) signifies the volume of the selected sample solution at the adsorption time *t*, and *M* (g) represents the weight of the magnetic hemicellulosic microspheres. This equation allows for the quantification of the Cu^2+^ adsorption amount in relation to the concentration of copper ions over time.

#### 2.4.2. Effect of pH on the Adsorption Amount

The acidity of the solution is essential for adsorption, affecting both the adsorption sites on the adsorbent and the physicochemical state of heavy metal ions. A notable relationship exists between the adsorption capacity of the microsphere and the pH of the solution. The pH affects the surface charge distribution of the magnetic composite microspheres as well as the degree of protonation and the state of copper ions present. To explore how pH impacts the removal of copper ions from the solution, pH values from 2.0 to 6.0 were chosen for this study. A standard Cu^2+^ solution was prepared by diluting from 1 mg/mL to 60 μg/mL. The diluted solution (600 mL) was evenly distributed into six 100 mL Erlenmeyer flasks, with the pH adjusted to 2.0, 3.0, 4.0, 5.0, and 6.0 using small amounts of hydrochloric acid (3 wt.%) and ammonia (20%, *v/v*). To each flask, 20 mg of magnetic hemicellulose microspheres were added, and the flasks were placed in a water bath at 40 °C, stirring for 6 h. After allowing the mixture to stand and then centrifuging, the supernatant was analyzed to measure the concentration of Cu^2+^. A graph was created with the amount of adsorption (q) plotted on the ordinate and pH on the abscissa.

#### 2.4.3. Effect of Initial Cu^2+^Concentration on the Adsorption Amount

To investigate the impact of initial Cu^2+^ concentration, a standard Cu^2+^ solution was diluted from 1 mg/mL to concentrations of 40 μg/mL, 50 μg/mL, 60 μg/mL, 70 μg/mL, and 80 μg/mL. These diluted solutions were then added to five 100 mL Erlenmeyer flasks, with the pH adjusted to 5.0 using a small amount of ammonia (20%, *v*/*v*). Each flask contained 20 mg of magnetic hemicellulose microspheres and was placed in a constant temperature shaker at 40 °C for 24 h. After allowing the magnetic microspheres to settle using a magnet, the supernatant was analyzed for Cu^2+^ concentration. A graph was created with the amount of adsorption (q) plotted on the ordinate and the initial concentration of Cu^2+^ on the abscissa.

#### 2.4.4. Effect of Adsorption Time on the Adsorption Amount

A standard Cu^2+^ solution was diluted from 1 mg/mL to 60 μg/mL. This diluted solution was transferred to a 100 mL Erlenmeyer flask, and the pH was adjusted to 5.0 using ammonia (20%, *v/v*). Then, 20 mg of magnetic hemicellulose microspheres was added to the flask, which was then placed in a constant temperature shaker at 40 °C for 24 h. Sampling was conducted at intervals of 0.5 h, 1 h, 2 h, 4 h, 6 h, 8 h, 10 h, 12 h, and 24 h. A magnet was employed to isolate the magnetic hemicellulose microspheres, and the supernatant was analyzed for Cu^2+^ concentration. A graph was created with the amount of adsorption (q) plotted on the ordinate and the adsorption time as abscissa.

#### 2.4.5. Reusability of the Microspheres

To assess the reusability properties, a diluted hydrochloric acid solution (0.1 mol/L) was utilized as the desorption agent for the magnetic hemicellulosic microspheres that had reached adsorption equilibrium in an 80 μg/mL Cu^2+^ solution. The desorption process was conducted in a constant temperature oscillator at room temperature for 24 h. After desorption, the microspheres were separated, washed, lyophilized, and reused. The magnetic hemicellulosic microspheres was reused for three times. Ultimately, the retention rate of the adsorption capacity of the microspheres was measured, enabling an evaluation of their recycling performance.

## 3. Results and Discussion

### 3.1. TEM Analysis of Fe_3_O_4_ Nanoparticles

The Fe_3_O_4_ nanoparticles were synthesized using the coprecipitation method and then ultrasonically dispersed in deionized water. The resulting suspension was deposited onto a copper grid. After the water evaporated, the grid was analyzed with transmission electron microscopy (TEM), as shown in Figure S1. The analysis clearly shows that the Fe_3_O_4_ nanoparticles predominantly have a spherical shape, with sizes ranging from 10 to 15 nm.

### 3.2. Characterization of the Microsphere

#### 3.2.1. Relationship Between Particle Size of Microspheres and Preparation Progress Parameters

There was a notable relationship between the average particle size of the magnetic hemicellulosic microspheres and the parameters used in the process. By adjusting the ratio of the oil and water phases, the amount of dispersing agent, and the stirring speed, the average particle size of the microspheres can vary from 20 μm to 1863 μm. As indicated in Table 1, increasing the stirring speed, the oil-to-water phase ratio, and the amount of dispersant led to a reduction in the particle size of the microspheres.

#### 3.2.2. Analysis of FT-IR Spectrum

**Figure 3:**
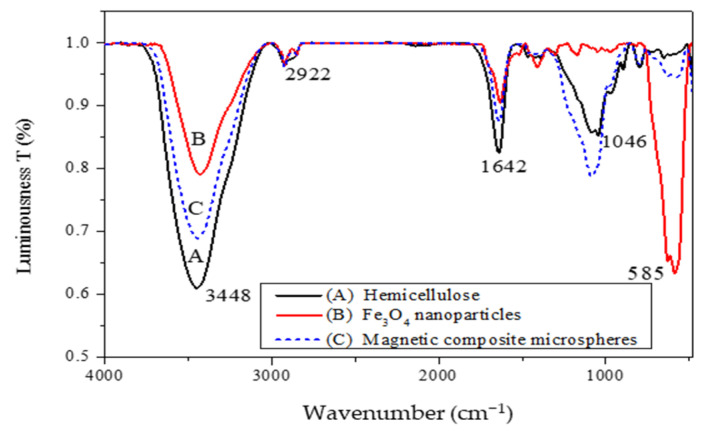
Infrared spectra: (A) hemicellulose; (B) Fe_3_O_4_ nanoparticles; (C) magnetic composite microspheres.

Figure 3 shows the infrared spectrograms of hemicellulose (A), Fe_3_O_4_ nanoparticles (B), and magnetic hemicellulosic microspheres (C, sample 4). The spectral peak positions for A and C are nearly identical, indicating that the primary functional groups of hemicellulose remained intact during the creation of the magnetic composite microspheres. This suggested that hemicellulose was dissolved in the ionic liquid without undergoing any derivatization reactions. The peaks at 3448 cm^−1^ and 2922 cm^−1^ correspond to the stretching vibrations of O-H and C-H, respectively, while the peak at 1642 cm^−1^ is linked to water adsorption. In spectrum A, a significant adsorption peak at 1046 cm^−1^ is recognized as the characteristic peak of xylan, resulting from the stretching vibrations of C-O, C-C, and the vibrations of C-O-C in glycosidic bonds. In spectrum B, the absorption peak at 585 cm^−1^ is related to Fe_3_O_4_, attributed to the Fe-O group. This characteristic absorption peak is also present in spectrum C, confirming the existence of Fe_3_O_4_ nanoparticles in the sample. This indicates that the magnetic hemicellulose/Fe_3_O_4_ microspheres have been successfully synthesized. Spectrum C was a result of the magnetic hemicellulosic microspheres comprised of hemicellulose and Fe_3_O_4_ nanoparticles, and the content of hemicellulose in microspheres was low in comparison with hemicellulose sample (A). Therefore, the peak intensity of hemicellulose in spectrum C is lower than that in spectrum A [52,53]. Otherwise, the used ionic liquid may reduce the hydrogen bond interactions within hemicellulose, and there is an electrostatic interaction between the negatively charged surface of hemicellulose and the Fe_3_O_4_ nanoparticles. Furthermore, the hydroxyl groups in hemicellulose could promote the formation of hydrogen bonds with the Fe_3_O_4_ nanoparticles.

#### 3.2.3. SEM and TEM Analysis

**Figure 4:**
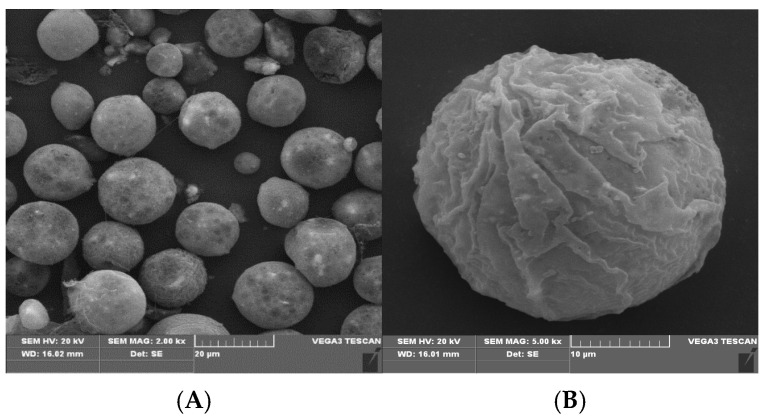
SEM images of magnetic hemicellulosic microspheres: (**A**) 2000× magnification; (**B**) 5000× magnification.

**Figure 5:**
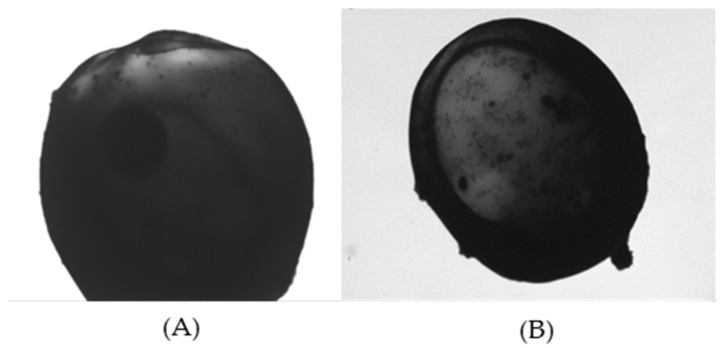
TEM photographs of magnetic hemicellulosic microspheres: (**A**) 15,000× magnification; (**B**) 20,000× magnification.

Figure 4 shows the scanning electron microscopy (SEM) of the magnetic hemicellulosic microspheres (sample 4). As shown in the image A (2000× magnification), the microspheres have a spherical shape, with an average particle size of about 20 μm. The laser particle size analyzer also showed that the average particle size of the microspheres (sample 4) was 20 μm in Table 1. The image B (5000× magnification) clearly illustrates that the surface of the microspheres is rough, featuring numerous wrinkles and pores. This surface structure contributes to a significant specific surface area and improved adsorption properties, making the microspheres ideal for various functional applications [54]. During the preparation, hemicellulose is restructured from the ionic liquid through both intramolecular and intermolecular hydrogen bonding. The Fe_3_O_4_ nanoparticles can easily attach to the hemicellulose by forming hydrogen bonds with its hydroxyl groups. Moreover, the negative charge on the surfaces of both the hemicellulose and Fe_3_O_4_ nanoparticles can promote electrostatic interactions, which aid in stabilizing the formation of composite microspheres. Figure 5 presents the transmission electron microscopy (TEM) of the magnetic hemicellulosic microspheres. The A and B images revealed that the Fe_3_O_4_ nanoparticles were distributed throughout the composite microspheres. The wrinkles on the surface, as seen in Figure 4, further confirmed that the Fe_3_O_4_ nanoparticles had been successfully integrated into the hemicellulosic matrix.

#### 3.2.4. Analysis of Granularity

**Figure 6:**
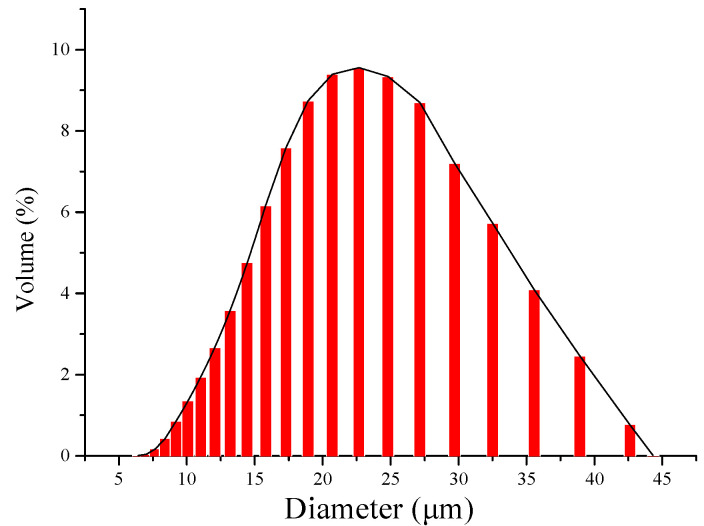
Particle size distribution of magnetic hemicellulosic microspheres.

Figure 6 shows the particle size distribution of the magnetic hemicellulosic microspheres, indicating that most microspheres fall within the 20 to 25 μm range, which aligns with the SEM findings.

#### 3.2.5. Analysis of VSM Results

**Figure 7:**
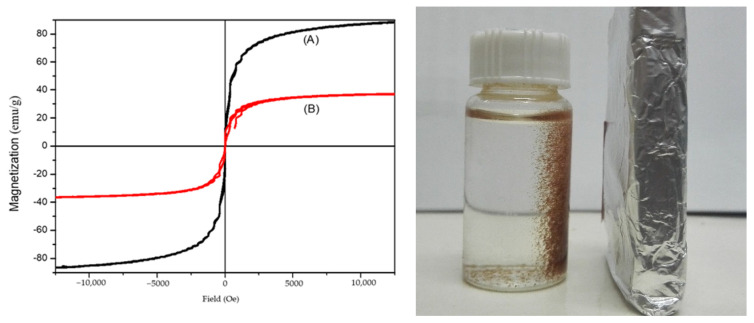
Magnetization curves: (A) Fe_3_O_4_ nanoparticles; (B) magnetic hemicellulosic microspheres.

Figure 7 displays the magnetization curves for both Fe_3_O_4_ nanoparticles (A) and the magnetic hemicellulosic microspheres (B). The saturation magnetization values were recorded at 88.07 emu/g for the Fe_3_O_4_ nanoparticles and 35.95 emu/g for the magnetic hemicellulosic microspheres. Importantly, the magnetization curve of the Fe_3_O_4_ nanoparticles shows no residual magnetism or coercive force, indicating superparamagnetic behavior. Figure 7 also shows a photograph of the magnetic hemicellulosic microspheres being attracted to a magnet. The microspheres quickly respond to the magnetic field, aligning with it within 3 s. This characteristic implies that the magnetic composite microspheres can be easily gathered and separated from the reaction system using a magnetic field, which is especially advantageous for applications in heavy metal removals from waste water.

### 3.3. Adsorption Study of Copper Ions by the Microspheres

Figure S2 shows the standard analysis curve for Cu^2+^ ions. The linear regression equation obtained from the data is given by Equation (2), as follows:

(2) $$C=\frac{A+0.0022}{0.0065}$$

Here, *C* (μg/mL) represents the concentration of the Cu^2+^ solution, and *A* is the absorbance value. The correlation coefficient *R*^2^ of 0.9993 indicates a strong linear relationship. Within the concentration range of 5.0 μg/mL to 30 μg/mL, there is a clear linear correlation between the concentration of Cu^2+^ (C) and the absorbance (A).

#### 3.3.1. Effect of pH on the Adsorption Property of the Microspheres

Figure S3 illustrates the pH influence on the adsorption capacity of the microspheres for Cu^2+^ ions. The adsorption capacity of the microspheres significantly increased with the rising pH, reaching its maximum at a pH of 5.0, after which it starts to decline. This trend can be explained by the complex interactions between the hydroxyl groups in the magnetic hemicellulosic microspheres and copper ions, which enhance the chemisorption of copper ions onto active sites. However, the active hydroxyl groups may become protonated in aqueous solutions, competing with the complex reaction. As the pH decreases, the concentration of hydrogen ions in the solution rises, promoting the protonation of the active hydroxyl groups and thus reducing complexity. When the pH goes above 5.0, an alkaline pH-adjusting agent should be added, since the aqueous solution of copper sulfate is weakly acidic. This can cause some copper ions to precipitate with the alkaline agent, which in turn lowers the adsorption capacity of the magnetic hemicellulosic microspheres [55,56]. In summary, the optimal pH value for the adsorption of copper ions by the magnetic hemicellulosic composite microspheres was 5.0.

#### 3.3.2. Effect of Initial Cu^2+^ Concentration on the Adsorption Property of the Microspheres

Figure S4 illustrates how the initial Cu^2+^ concentration affects the adsorption capacity of the microspheres. At lower initial Cu^2+^ concentrations, the adsorption amount of the copper ions by the microspheres was also low, likely due to the microspheres not being fully saturated. As the initial Cu^2+^ concentration increased, the adsorption amount rose, peaking at 85.65 mg/g at 323 K. This increase was due to the greater concentration gradient between the Cu^2+^ ions in the solution and the active sites on the microsphere surfaces. According to Fick’s law, this gradient boosts the diffusion flux and the driving force for adsorption, promoting the interaction between the active sites on the microspheres and Cu^2+^ ions. However, as more active sites become occupied during the adsorption process, saturation occurs, leading to a maximum adsorption amount [57].

#### 3.3.3. Effect of Adsorption Time on the Adsorption Capacity of the Microspheres

Figure S5 shows the impact of adsorption time on the copper ion adsorption on the microspheres. As the adsorption time increased, the Cu^2+^ adsorption amount by the magnetic hemicellulosic microspheres continued to grow until the adsorption equilibrium was achieved. At this stage, the Cu^2+^ adsorption amount was recorded at 75.24 mg/g. The adsorption process unfolded in the following two distinct phases: during the first 0 to 2 h, the adsorption amount rose quickly, accounting for nearly 60% of the total adsorption amount; after 2 h, the rate of adsorption slowed, with 97% of the total adsorption completed by 12 h. After this period, the amount of adsorption leveled off, indicating that the adsorption equilibrium has been reached. The rapid adsorption phase mainly took place on the surface of the magnetic hemicellulosic microspheres, where a high number of active sites and a significant concentration gradient of Cu^2+^ ions enabled swift and reversible adsorption. As the adsorption continued, the concentration of copper ions diminished, and the number of available active sites decreased. This led to greater resistance to adsorption, causing a decline in the adsorption rate until the equilibrium was eventually reached [57,58].

### 3.4. Adsorption Kinetics of the Microspheres

To explore how the magnetic hemicellulosic microspheres adsorb copper ions, the following four different adsorption kinetics models were used to analyze the obtained experimental data: the pseudo-first-order kinetic model [59], the pseudo-second-order kinetic model [60], the intraparticle diffusion model [61], and Elovich’s kinetic model [62]. The corresponding Equations (3)–(6) for these models are as follows:

(3) $$\mathrm{lg}(q_{e}-q_{t})=\mathrm{lg}q_{e}-\frac{k_{1}}{2.303}t$$

(4) $$\frac{t}{q_{t}}=\frac{1}{k_{2}{q_{e}}^{2}}+\frac{1}{q_{e}}t$$

(5) $$q_{t}=K_{p}t^{1/2}+C$$

(6) $$q_{t}=\frac{1}{b}\ln(ab)+\frac{1}{b}\ln t$$

In these equations, *q_e_* (mg/g) and *q_t_* (mg/g) indicate the adsorption amounts at adsorption equilibrium and at time *t*, respectively; *t* represents the adsorption time; and *k_1_* (min^−1^) and *k_2_* (g/(mg·min)) are the rate constants for the pseudo-first and pseudo-second order kinetics. The parameters *a* (mg/(g·min)) and *b* (mg/g) denote the initial rate of adsorption and Elovich’s parameter, respectively, which relates to the extent of surface coverage of the adsorbent and the energy involved in chemisorption.

Figure S6 shows the mathematical curve fitting of the experimental data for the four adsorption kinetics models, and the fitting parameters are listed in Table 2. By examining the correlation coefficients *R*^2^ of the fitting curves, the pseudo-second-order kinetic model, which had an *R*^2^ value of 0.993, was closest to 1. This indicated that the adsorption of copper ions onto the magnetic hemicellulosic microspheres followed the pseudo-second-order equation. The pseudo-second-order kinetic model could characterize the adsorption process as being influenced by the concentration gradient, which affected the diffusions of copper ions to the microspheres and the interactions of copper ions with active sites until the saturation was reached. This suggested that the adsorption reaction was driven by the concentration factor, with the formation of chemical bonds being the main mechanism, thus confirming that chemical adsorption was the prevailing process.

### 3.5. Adsorption Thermodynamics of the Microspheres

To better understand how copper ions were adsorbed by the magnetic hemicellulosic composite microspheres, in this study, the Langmuir [63] and Freundlich adsorption isotherm models were applied [64] to analyze the experimental data on adsorption. Equations (7) and (8) of the Langmuir and Freundlich adsorption isotherms are as follows:

(7) $$\frac{C_{e}}{q_{e}}=\frac{C_{e}}{q_{\max}}+\frac{1}{K_{L}q_{\max}}$$

(8) $$\mathrm{lg}q_{e}=\mathrm{lg}K_{F}+\frac{1}{n}\mathrm{lg}C_{e}$$

In the two equations, *C_e_* (mg/L) refers to the residual concentration of Cu^2+^ in the solution at adsorption equilibrium; *q_e_* (mg/g) indicates the adsorption capacity at this equilibrium; and *q_max_* (mg/g) signifies the theoretical maximum adsorption capacity according to the Langmuir model. *K_L_* represents the strength of adsorption in the Langmuir model, reflecting the affinity between the sorbent and the adsorbate. Additionally, *n* and *K_F_* (mg^1−n^ g^−1^ L^−n^) are the constants associated with the Freundlich model.

The fitting parameters of the two adsorption isotherm models, shown in Table 3, indicate that at the temperatures of 303 K, 313 K, and 323 K, the Langmuir isotherm model offers a better fit for the experimental data. This suggests that the adsorption of copper ions onto the magnetic composite microspheres mainly adhered to the Langmuir isotherm model, implying that monolayer adsorption was the key mechanism. The constant *K_L_*, which is the adsorption equilibrium constant, represents the inherent characteristics of both the adsorbent and adsorbate, as well as the temperature of the system, and it has a positive correlation with the adsorption capacity [65]. The findings showed that when other conditions remained unchanged, an increase in temperature led to a higher *K_L_* value, thus improving the adsorption capacity.

The Freundlich parameter *n* values in Table 3, all exceeding 1, indicated that copper ions were readily adsorbed by the magnetic hemicellulosic microspheres. Additionally, the high correlation coefficients for the Freundlich isotherm indicated that the adsorption of copper ions from the aqueous solution involved both physical adsorption and chemisorption mechanisms.

To further investigate the adsorption behavior of the magnetic hemicellulosic microspheres, the simplified expression was used to describe the essential features of Langmuir adsorption isotherm followed by Equation (9):

(9) $$R_{L}=\frac{1}{1+C_{0}K_{L}}$$

Here, *R_L_* is a dimensionless parameter; *C_0_* (mg/L) is the initial concentration of Cu^2+^; and *K_L_* is the model parameter of the Langmuir adsorption isotherm.

As listed in Table 4, all *R_L_* values were within the range of 0 to 1, indicating that the copper ion adsorption on magnetic hemicellulosic microsphere is a spontaneous process [66].

### 3.6. Reusability Performance of the Microspheres

**Figure 8:**
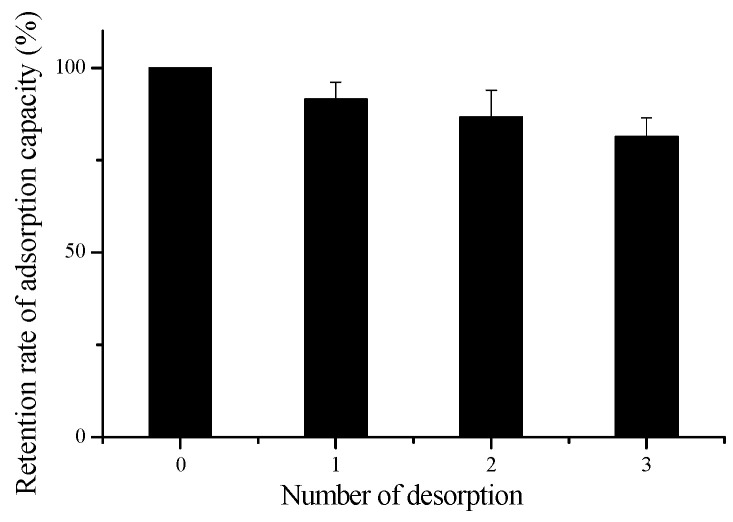
Desorption results of the magnetic hemicellulosic microspheres.

Figure 8 shows the results of the reusability of magnetic hemicellulosic microspheres in adsorbing copper ions. After undergoing three cycles of desorption and adsorption, it is found that the microspheres maintained 81.36% of their adsorption capacity for copper ions, demonstrating its good recycling potential [67], and previous studies using natural polymers-based hydrogels also indicated similar reuse percentages, 80% for lignin-g-PAAc/MMT hydrogel [68], and 85.3% for straw biopolymer-based hydrogel [69]. The magnetic hemicellulosic microspheres discussed in this research represent a novel type of polymer material with outstanding properties.

## 4. Conclusions

In this study, the hemicellulose extracted from wheat straw was used to prepare magnetic hemicellulosic microspheres. Various characterization techniques were applied to investigate their properties, including the structure, morphology, and magnetic features. Fe_3_O_4_ nanoparticles were synthesized through the coprecipitation method, and the hemicellulosic microspheres were prepared by a direct synthesis method using the ionic liquid B[mim]Cl as a solvent of hemicellulose. This ionic liquid facilitated the dissolution of hemicellulose, allowing for the effective distribution of Fe_3_O_4_ nanoparticles within the hemicellulose solution.

This research discovered an efficient way of creating hemicellulosic magnetic microspheres as well as the process parameters required, and the microspheres are suitable for heavy metal adsorption. The average diameter of the microspheres, whose surface was rough and irregular with wrinkles and pores, was around 20 μm. The Fe_3_O_4_ nanoparticles were uniformly encapsulated within the microspheres, which displayed a saturation magnetization of 35.95 emu/g. Under optimal adsorption conditions (pH = 5.0, T = 323 K, C_0_ = 80 g/L), the maximum adsorption capacity was 85.65 mg/g over a 24 h period. In this study, it was found that the adsorption of copper ions onto the magnetic hemicellulosic microspheres followed the pseudo-second-order kinetic model and the Langmuir isotherm model, which suggested that the adsorption process was governed by chemical adsorption, with a monomolecular layer forming on the surface of the microspheres. The Langmuir equation gave a theoretical value of a maximum adsorption capacity of 149.25 mg/g. In summary, the magnetic hemicellulosic microspheres with high adsorption capacity and effective performance were synthesized for the removal of copper ions from water.

Future studies would involve the investigation of potential applications of the magnetic hemicellulosic microspheres for the removal of other heavy metals and pollutants present in wastewater, which would extend the spectrum of contaminants removal. Additional research could also study the stability of the microspheres, over more than three adsorption cycles to determine long-term effectiveness. Further research should also aim to optimize the synthesis process to minimize the cost and increase the scalability toward industrial settings. Most importantly, by embedding these microspheres into filtration or column systems to achieve continuous water treatment, it may be a direction for practical use in real-life engineering, to better achieve the application of large-scale copper ion recycling and removal.

## Figures and Tables

**Table 1:** Relation between particle sizes of microspheres and preparation progress parameters.

Sample	Stirring Speed (r/min)	Oil/Water (*v*/*v*)	Dispersant (g)	Average Particle Size (μm)
1	300	2:1	1	1360
2	600	3:1	2	390
3	900	4:1	3	110
4	1000	5:1	4	20

**Table 2:** Fitting parameters of different adsorption kinetics models.

Pseudo-First-Order Kinetic Model			Pseudo-Second-Order Kinetic Model			Intra-Particle Diffusion Model			Elovich’s Kinetic Model		
*q_e_* (mg/g)	*k*_1_ (min^−1^)	*R* ^2^	*q_e_* (mg/g)	*k*_2_ (g/(mg·min))	*R* ^2^	*K_p_* (mg/(g·min^0.5^))	*C* (mg/L)	*R* ^2^	*a* (mg/(g·min))	*b* (mg/g)	*R* ^2^
66.41	0.266	0.962	82.65	0.006	0.993	18.75	11.77	0.935	100.99	0.059	0.971

**Table 3:** Fitting parameters of the Langmuir and Freundlich adsorption isotherm models.

	Langmuir Adsorption Isotherm Model			Freundlich Adsorption Isotherm Model		
*T* (K)	*K_L_* (L/mg)	*q_max_* (mg/g)	*R* ^2^	*K_F_* (mg^1−n^ g^−1^L^−n^)	*n*	*R* ^2^
303	0.0212	133.33	0.9749	8.316	1.858	0.9601
313	0.0219	140.85	0.9818	9.076	1.874	0.9669
323	0.0230	149.25	0.9870	10.174	1.902	0.9710

**Table 4:** *R_L_* under different concentrations.

*C_0_* (μg/mL)	40	50	60	70	80
*R_L_* (303 K)	0.541	0.485	0.440	0.403	0.371
*R_L_* (313 K)	0.533	0.477	0.432	0.395	0.363
*R_L_* (323 K)	0.521	0.465	0.420	0.383	0.352

## Data Availability

Data are contained within the article and supplementary materials.

## Supplementary Materials

The following supporting information can be downloaded at: https://www.mdpi.com/article/10.3390/polym16243460/s1, Figure S1: TEM photograph of Fe_3_O_4_ nanoparticles in aqueous phase; Figure S2: standard analysis curve of Cu^2+^ ions; Figure S3: effect of pH on the adsorption capacity of the microspheres (microsphere dosage: 0.2 g/L, Cu^2+^ concentration: 60 mg/L, temperature: 313 K); Figure S4: effect of initial concentration of Cu^2+^ on the adsorption property of the microspheres (microsphere dosage: 0.2 g/L, time: 24 h); Figure S5: effect of adsorption time on the adsorption amount of copper ions; Figure S6: the fitting curves of four adsorption kinetic models: (**A**) pseudo-first-order kinetic model; (**B**) pseudo-second-order kinetic model; (**C**) intra-particle diffusion model; (**D**) Elovich’s kinetic model; Figure S7: the fitting curves of adsorption dynamic models: (**A**) Langmuir adsorption isotherm model (303 K, 313 K, 323 K); (**B**) Freundlich adsorption isotherm model (303 K, 313 K, 323 K).
