# Supplementary material for: Preparation of Magnetic Hemicellulosic Composite Microspheres and Adsorption of Copper Ions
Source: Polymers (Basel). 2024 Dec 11;16(24):3460. doi: 10.3390/polym16243460 (PMC11679899; doi:10.3390/polym16243460)
Supplement: Supplementary file 1 [file polymers-16-03460-s001.zip › polymers-3339367-supplementary.pdf]

polymers

MDPI

Supplementary materials for

Preparation of magnetic hemicellulosic composite microspheres and adsorption of copper ions

Muhammad Sheraz[1], Xiao-Feng Sun[1,2,*], Yong-Ke Wang[1], Adeena Siddiqui[3], Jiayi Chen[1], Le Sun[1]

1 Research Centre of Advanced Chemical Engineering, School of Chemistry and Chemical Engineering, Northwestern Polytechnical University, Xi'an, 710129, China
2 Shenzhen Research Institute, Northwestern Polytechnical University, China
3 Shaheed Zulfikar Ali Bhutto Institute of Science and Technology University, Karachi, Pakistan
* Correspondence: E-mail address: xf001sn@nwpu.edu.cn (X.F. Sun).

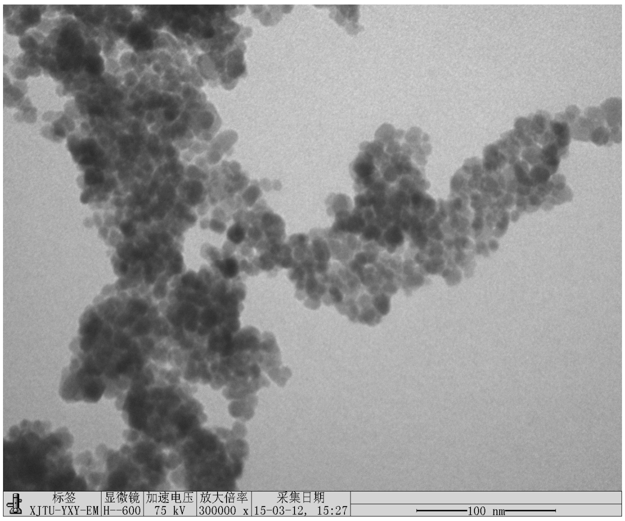

Figure S1. TEM photograph of Fe_3O_4 nanoparticles in aqueous phase.

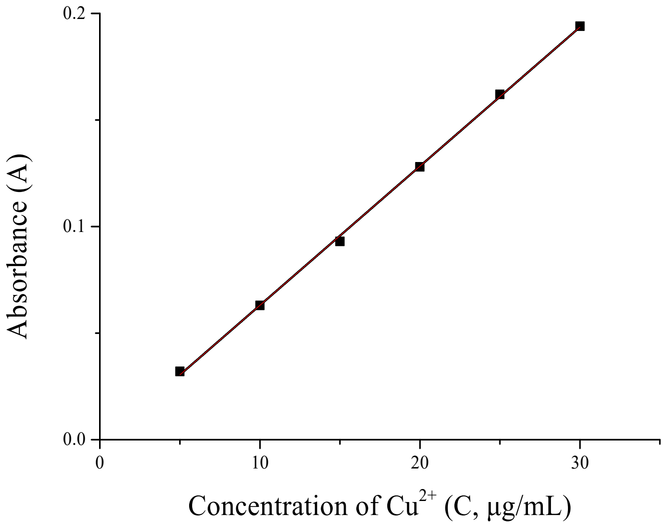

Figure S2. Standard analysis curve of Cu^{2+} ions concentration.

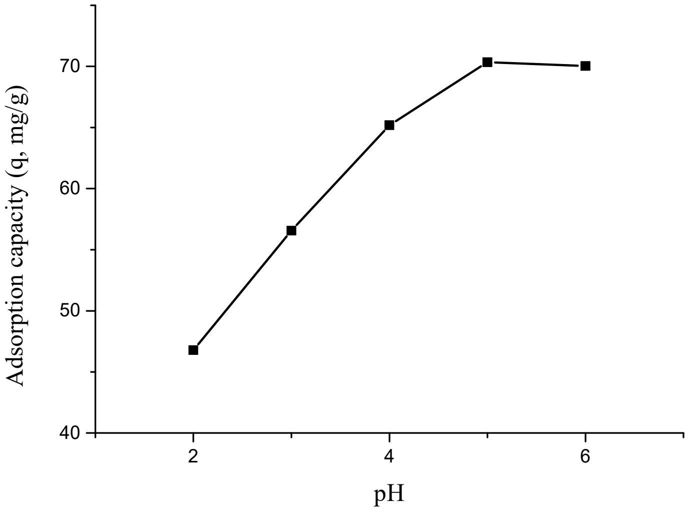

Figure S3. Effect of pH on the adsorption capacity of the microspheres (Microspheres dosage: 0.2 g/L, Cu^{2+} concentration: 60 mg/L, temperature: 313 K).

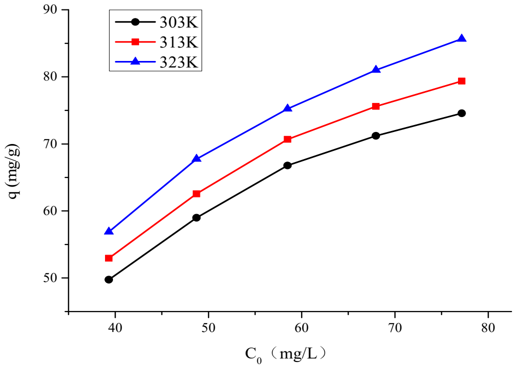

Figure S4. Effect of initial concentration of Cu^{2+} on the adsorption property of the microspheres (Microspheres dosage: 0.2 g/L, time: 24 h, temperature: 313 K).

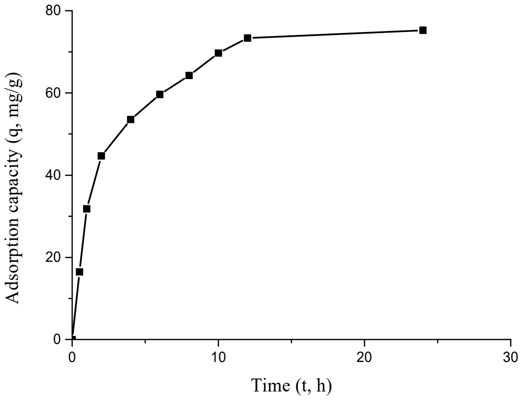

Figure S5. Effect of adsorption time on the adsorption amount of copper ions (Microspheres dosage: 0.2 g/L, Cu^{2+} concentration: 60 mg/L, temperature: 313 K).

.

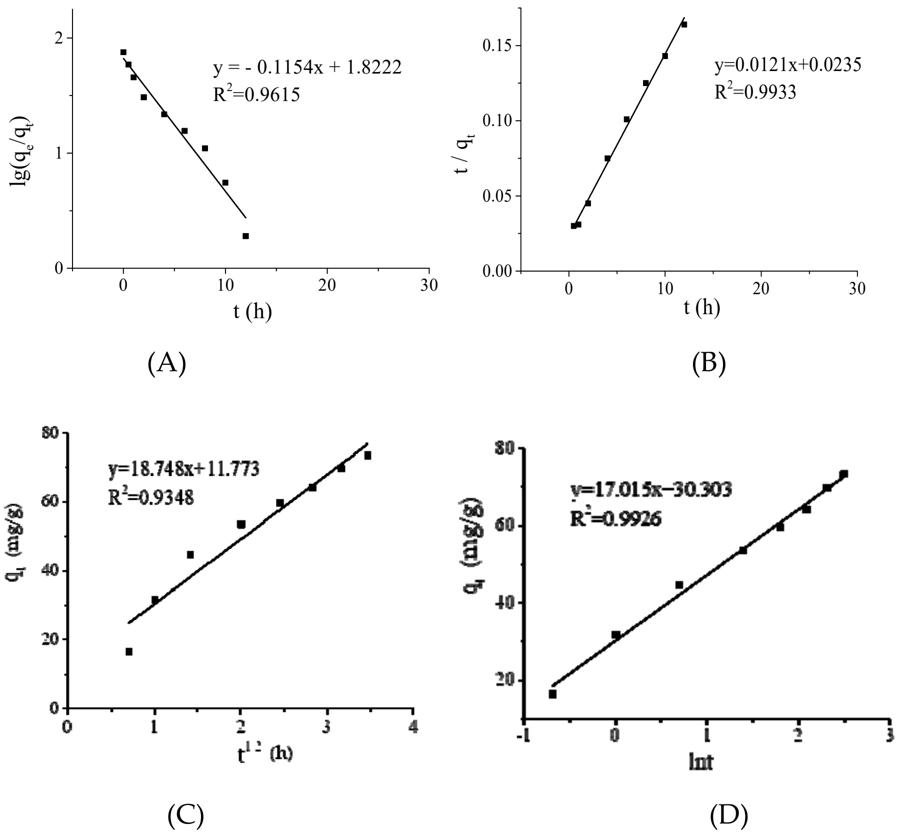

Figure S6. The fitting curves of four adsorption kinetic models: (A) Pseudo-first-order kinetic model; (B) Pseudo-second-order kinetic model; (C) Intra-particle diffusion model; (D) Elovich's kinetic model.

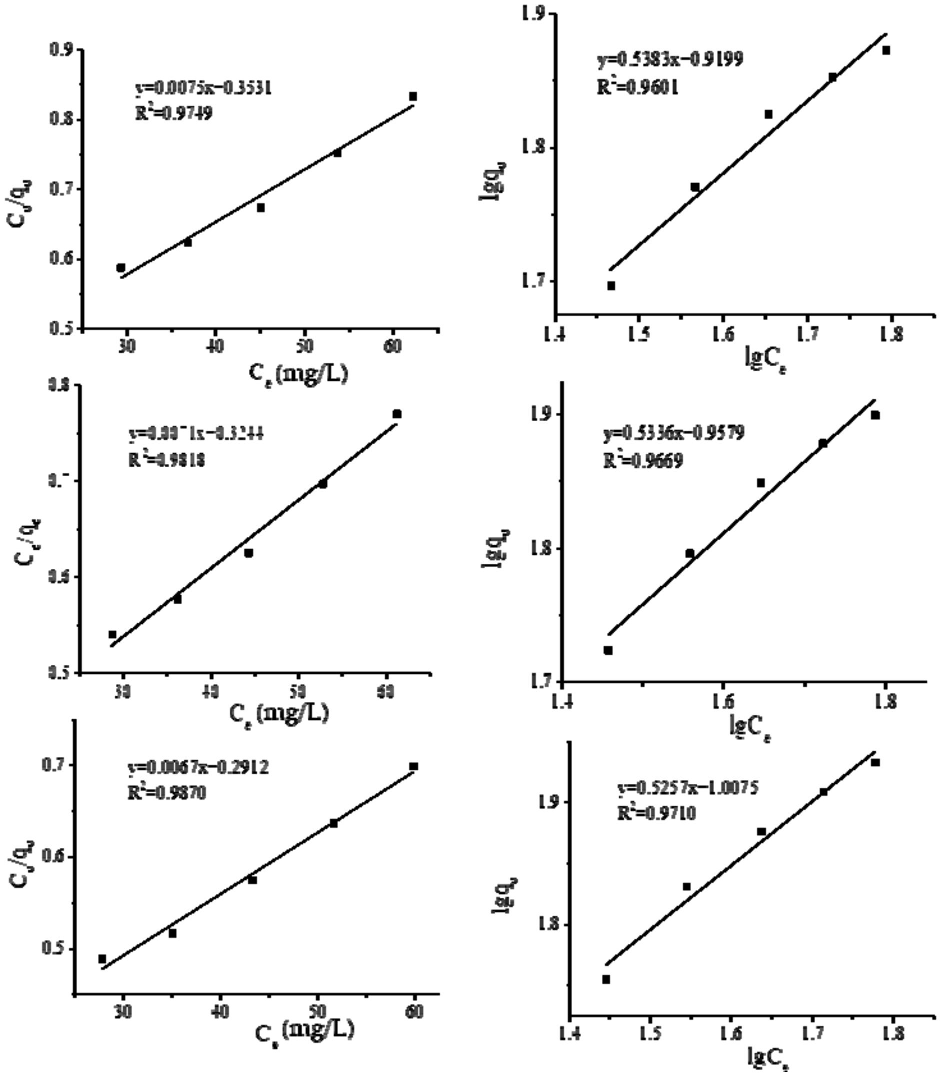

（A） （B）

Figure S7. The fitting curves of adsorption dynamic models: (A) Langmuir adsorption isotherm model (303 K, 313 K, 323 K); (B) Freundlich adsorption isotherm model (303 K, 313 K, 323 K).

Disclaimer/Publisher's Note: The statements, opinions and data contained in all publications are solely those of the individual author(s) and contributor(s) and not of MDPI and/or the editor(s). MDPI and/or the editor(s) disclaim responsibility for any injury to people or property resulting from any ideas, methods, instructions or products referred to in the content.
